# Review of the Oriental Monotypic Genus *Pibrocha* Kirkaldy (Hemiptera, Fulgoromorpha, Fulgoridae, Dorysarthrinae)

**DOI:** 10.3897/zookeys.132.1319

**Published:** 2011-10-03

**Authors:** Zhi-Shun Song, Thierry Bourgoin, Ai-Ping Liang

**Affiliations:** 1 Key Laboratory of the Zoological Systematics and Evolution, Institute of Zoology, Chinese Academy of Sciences, No. 1 Beichen West Road, Chaoyang District, Beijing 100101, China; 2 Département Systématique & Evolution, UMR 7205 MNHN-CNRS, Muséum National d’Histoire Naturelle. Case Postale 50 / Entomologie, 45, Rue Buffon, F-75231 Paris cedex 05, France

**Keywords:** Fulgoridae, Dictyopharidae, Dorysarthrinae, *Pibrocha*, redescription

## Abstract

The monotypic genus *Pibrocha* Kirkaldy, 1902, known only from Sri Lanka in the Oriental region, is closely related to *Dorysarthrus* Puton, 1895 from southwestern Asia and northern Africa (Palaearctic region). The genusis revised to include a first description of the male genital structures and a discussion of relationships between *Pibrocha*, *Dorysarthrus* and *Dichoptera* Spinola, 1839. A diagnostic key to the three genera and photos of their type species are provided for better comparison in these taxa. *Pibrocha* is assigned tentatively from Dictyopharidae to the subfamily Dorysarthrinae (Fulgoridae).

## Introduction

The monotypic genus *Pibrocha* was established by Kirkaldy (1902) to accommodate a peculiar species *Dictyophora* [sic] *egregia* Kirby, 1891 from Sri Lanka. Kirkaldy (1902) stated that “the genus is closely allied to *Dictyophara* Germar, but, beyond other differences, is readily recognizable by the transverse nervure in the clavus, which thus allies it to *Dichoptera* Spin. (Kirkaldy 1902: 51).”

Traditionally, the genus *Pibrocha*, along with other genera *Awaramada* Distant, 1914, *Daridna* Walker, 1858, *Dichoptera* Spinola, 1839 and *Dorysarthrus* Puton, 1905, was placed in the subtribe Dichopterina (Dictyopharidae: Dictyopharinae: Dichopterini) for the presence in the forwings of a short claval crossvein between CuP and Pcu (Metcalf 1946). Among these genera, *Daridna* was transferred to the leafhopper family Cicadellidae by Nielson (1982) and *Awaramada* was synonymized with *Pibrocha* by Liang (2000).

While attempting to clarify the distinction between Fulgoridae and Dictyopharidae, Emeljanov (1979) regarded the short crossvein in the clavus as one of familial diagnostic characters. Emeljanov (1979) elevated Dichopterini (only *Dichoptera*) to subfamily status (Dichopterinae) and established a new monotypic subfamily Dorysarthrinae for *Dorysarthrus*. Both monotypic subfamilies were transferred by Emeljanov (1979), in company with some other dictyopharid taxa, to the lanternfly family Fulgoridae, which is widely accepted to be a sister group of Dictyopharidae in the hypotheses of Fulgoromorpha phylogeny based on either morphological characters or DNA sequence data (Asche 1987; Emeljanov 1990; Bourgoin 1993; Yeh et al. 2005; Urban and Cryan 2009). Thus only *Pibrocha* was not considered and its taxonomic status is not discussed until now.

**Figures 1–3.**
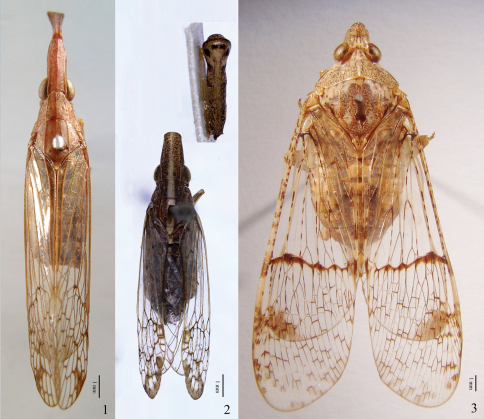
**1** *Pibrocha egregia* (Kirby), ♂, dorsal view **2** *Dorysarthrus mobilicornis* Puton, holotype ♀, dorsal view **3** *Dichoptera hyalinata* (Fabricius), ♂, dorsal view. Scale bars: Figs 1–3 = 1 mm.

**Figures 4–6.**
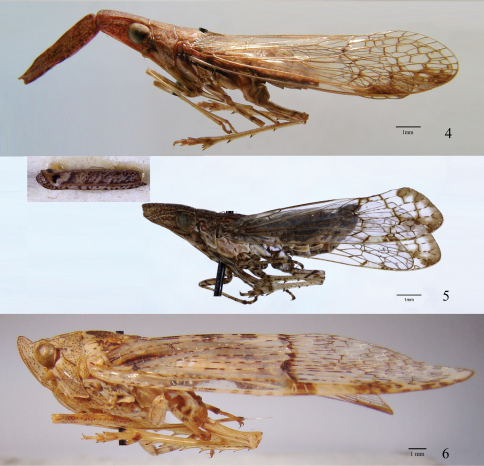
**4** *Pibrocha egregia* (Kirby), ♂, lateral view **5** *Dorysarthrus mobilicornis* Puton, holotype ♀, lateral view **6** *Dichoptera hyalinata* (Fabricius), ♂, lateral view. Scale bars: Figs 4–6 = 1 mm.

The speces *Pibrocha egregia* possesses a very elongate cephalic process, which is furrowed and constricted at its basal 1/3, and appears to be ‘fractured’ and separated into two portions by an articulation (Figs 1, 4). In many dead dried specimens, the distal portion of cephalic process is easily broken, so the species may be easily misidentified. As an example the monotypic genus *Awaramada* Distant was established based on *Pibrocha* specimen that had lost the distal portion of the cephalic process. Its type species *Awaramada fryeri* Distant, 1914 was synonymized with *Pibrocha egregia* by Liang based on examination of type material in the Natural History Museum, London, UK (BMNH) (Liang 2000).

This study provides a review of the genus *Pibrocha*, including a first description of the male genital structures and a discussion of relationships between *Pibrocha*, *Dorysarthrus* and *Dichoptera*. A key to three genera and photos of their type species are also provided for better comparison in these taxa. *Pibrocha* is assigned tentatively to the subfamily Dorysarthrinae (Fulgoridae) from Dictyopharidae.

## Materials and methods

The male genitalia were cleared in 10% KOH at room temperature for ca. 12 hours, rinsed in distilled H_2_O, then transferred to glycerol for examination.

Morphological characters were observed with a Zeiss (Stemi SV II) optical stereomicroscope and illustrated with the aid of a drawing tube; measurements were made with the aid of an eyepiece micrometer.

The specimens studied in the course of this work are deposited in the following institutions whose names are abbreviated in the text as follows:

BMNH the Natural History Museum, London, UK;

MNHN the Museum National d’Histoire Naturelle, Paris, France;

NCSU Department of Entomology Insect Collection, North Carolina State University, Raleigh, North Carolina, USA;

USNM the National Museum of Natural History, Washington, D.C., USA.

The morphological terminology used in this study follows Emeljanov (1988) for external morphology and venation of the forewings, Bourgoin and Huang (1990) for male genitalia.

## Taxonomy

### Key to the genera *Pibrocha*, *Dorysarthrus* and *Dichoptera*

**Table:** 

1	Body very large and stout (large-sized species), body length (including forewings) usually more than 25 mm; head distinctly short, produced in a short or moderately long cephalic process, which is only 1/4 to half as long as pronotum and mesonotum combined (Fig. 3); cephalic process with apical portion before eyes abruptly narrowing to conic and distinctly upturned (Fig. 6); forewings with M vein first branching to MA and MP veins near base, and MP vein branching to MP_1_ and MP_2_ veins near basal 1/5 or 1/4 before nodal line; Sc+R, M and CuA veins branching to dozens of accessory veins beyond nodal line in forewings (Song and Liang, in prep.)	*Dichoptera* Spinola (Dichopterinae)
–	Body relatively much smaller and slender (medium-sized species); head very elongate and distinctly stout, produced anteriorly into a cephalic process, which is about twice as long as pronotum and mesonotum combined; cephalic process stout and cylindrical at basal 1/3, and then suddenly furrowed and constricted, which looks like being fractured and separated into two portions by an articulation; the distal remainder 2/3 turned downwards in lateral view (Fig. 8); forewings with M vein only branching to MA and MP veins near middle before nodal line; Sc+R, M and CuA veins branching to less accessory veins beyond nodal line in forewings	2 (Dorysarthrinae)
2	Cephalic process with distal remainder 2/3 inflated and subcylindrical, which is rounded and bulbous apically in dorsal view (Fig. 2); basal 1/3 of vertex without median carina, along with a broad white median band extending over pronotum and mesonotum; frons nearly parallel before postclypeus; pronotum and mesonotum bicarinate in middle disc, lateral carinae barely visible and median carina absent; hind tibiae with 7 apical black-tipped spines	*Dorysarthrus* Puton
–	Cephalic process with distal remainder 2/3 mostly narrowed and laterally compressed, gradually expanded and dorsoventrally compressed near apex, which is truncate and clavate apically in dorsal view (Fig. 1); basal 1/3 of vertex with median carina distinct and complete; frons widest and obtusely expanded outwards before postclypeus; pronotum and mesonotum tricarinate in middle disc, median and lateral carinae distinct and complete; hind tibiae with 6 apical black-tipped spines	*Pibrocha* Kirkaldy

### Family Fulgoridae Latreille, 1820

**Subfamily Dorysarthrinae Emeljanov, 1979**

#### Genus Pibrocha

Kirkaldy, 1902

http://species-id.net/wiki/Pibrocha

Pibrocha Kirkaldy, 1902: 50; Melichar, 1903: 20; Distant, 1906: 240; Melichar, 1912: 22; Metcalf, 1946: 31. Type species: *Dictyophora* [sic] *egregia* Kirby, 1891;by original designation.
Awaramada Distant, 1914: 412; Distant, 1916: 27; Metcalf, 1946: 31. Type species: *Awaramada fryeri* Distant, 1914; by monotypy. Synonymised by Liang, 2000: 235.

##### Diagnosis.

Cephalic process twice as long as pronotum and mesonotum combined, furrowed and constricted at basal 1/3, where it appears to be ‘fractured’ and separated into two portions by an articulation; the distal remainder 2/3 mostly narrowed and laterally compressed, gradually expanded and dorsoventrally compressed near apex, which is truncate and clavate in dorsal view, and turned downwards in lateral view; vertex with basal 1/3 broad and moderately arched, median carina distinct and complete; the remainder 2/3 of vertex and frons without median carina; pronotum and mesonotum tricarinate, nearly parallel; forewings elongate and slender, nearly four times as long as broad; M vein only branching to MA and MP veins near front-middle before nodal line and firstly branched before Sc+R and CuA veins near middle; clavus with a short crossvein, connecting CuP with Pcu; legs narrow and moderately long; fore femora not flattened and dilated, hind tibiae with 6 apical black-tipped spines; aedeagus large and symmetrical, with a pair of long and slender endosomal processes extended dorsally; phallobase basally sclerotized and pigmented, without spine.

##### Redescription.

**Figures 7–16.**
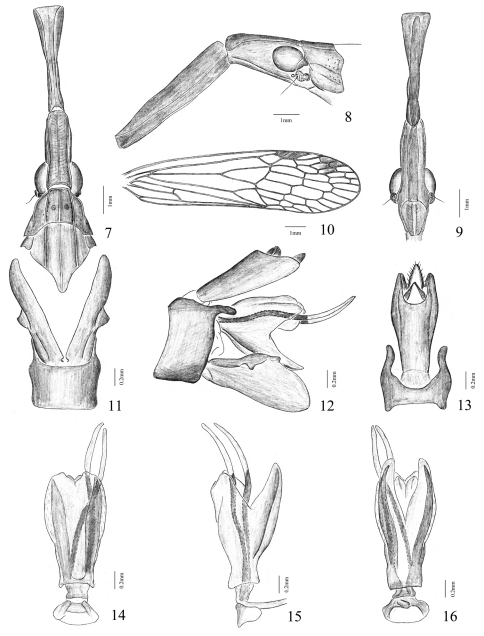
*Pibrocha egregia* (Kirby, 1891) **7** head, pronotum and mesonotum, dorsal view **8** head and pronotum, lateral view **9** head, ventral view **10** right forewing **11** pygofer and parameres of male, ventral view **12** genitalia of male, lateral view **13** pygofer and anal tube of male, dorsal view **14** aedeagus, dorsal view **15** aedeagus, lateral view **16** aedeagus, ventral view. Scale bars: Figs 7–10 = 1 mm, Figs 11–16 = 0.2 mm.

Head very elongate and distinctly stout, produced anteriorly into a cephalic process, which is about twice as long as pronotum and mesonotum combined. Cephalic process stout and cylindrical at basal 1/3, and then suddenly furrowed and constricted, where it appears to be ‘fractured’ and separated into two portions by an articulation; the distal remainder 2/3 mostly narrowed and laterally compressed, gradually expanded and dorsoventrally compressed near apex, which is truncate and clavate in dorsal view (Fig. 7), and turned downwards in lateral view (Fig. 8). Vertex with basal 1/3 broad and moderately arched, lateral carinae nearly sub-parallel and median carina distinct and complete; the remainder 2/3 narrowly sulcate, nearly parallel, gradually expanded and apically truncate, median carina indistinct in groove. Frons (Fig. 9) without median carina, intermediate carinae shallowly sulcate, nearly parallel; basal 1/3 widest and obtusely expanded outwards before postclypeus, lateral carinae slightly converging towards apex; the apical remainder 2/3 laterally compressed and abruptly narrowed. Postclypeus and anteclypeus convex medially, median carina indistinct. Rostrum long, reaching beyond abdominal segment V. Eyes oval and large. Ocelli large, reddish. Antennae with scape very small; pedicel large and subglobose, with more than 50 distinct sensory plaque organs distributed over entire surface; flagellum long, setuliform.

Pronotum (Fig. 7) a little shorter than mesonotum medially, narrow anteriorly, broad posteriorly; anterior margin slightly arched centrally, lateral marginal areas straight and sloping with two long lateral carinae on each side between eyes and tegulae, posterior margin very broadly concave; disc tricarinate in middle, median and intermediate carinae distinct and complete, with a big lateral pit at side of median carina, respectively. Mesonotum (Fig. 7) tricarinate in disc, nearly parallel. Forewings (Fig. 10) elongate and slender, nearly four times as long as broad; anterior and posterior margins more or less parallel, apex rounded; M vein only branching to MA and MP veins near front-middle before nodal line and firstly branched before Sc+R and CuA veins near middle; apical area with at least three rows of transverse veinlets, veinlets usually not aligned, but in each field running along its length; clavus with a short crossvein, connecting CuP with Pcu; stigma broad and distinct, with 3–5 cross veins. Legs narrow and moderately long; fore femora not flattened and dilated, hind tibiae with 4 lateral and 6 apical black-tipped spines; hind tarsomeres I with about 8–9 and tarsomeres II with about 6–7 black-tipped apical spines, respectively.

##### Distribution.

Sri Lanka.

#### Pibrocha egregia

(Kirby, 1891)

http://species-id.net/wiki/Pibrocha_egregia

Figs 1 4 7–16

Dictyophora [sic] *egregia* Kirby, 1891: 135, Pl. 5, Fig.4. Syntype[s] (?sex), Sri Lanka BMNH [not examined].
Pibrocha egregia (Kirby): Kirkaldy, 1902: 51, Pl. B, Fig. 2; Melichar, 1903: 21, Pl. I, Fig. 4; Distant, 1906: 240, Fig. 104; Melichar, 1912: 24, Pl. I, Fig. 10-12; Metcalf, 1946: 31.
Awaramada fryeri Distant, 1914: 413; Distant, 1916: 27, Fig. 14; Metcalf, 1946: 31. Holotype ♂, Sri Lanka (BMNH) [examined]. Synonymised by Liang, 2000: 235.

##### Redescription.

Male, narrow and elongate, body length (from apex of cephalic process to tip of forewings) 21.3–21.5 mm; length of head (including two portions: the former is from apex of cephalic process to curved part, the latter is from curved part to base of eyes) (3.2+5.5)–(3.3+5.4) mm, width (including eyes) 1.8 mm; length of forewings 11.8–12.5 mm.

Vertex, genae and frons dull brownish-ochraceous, speckled with fuscous, suffused with testaceous-red. Basal 1/3 of frons with some small fuscous spots between intermediate carinae and lateral carinae. Pronotum and mesonotum brownish-ochraceous, tens of punctate spots on each lateral area of pronotum fuscous. Thorax ventrally and legs pale ochraceous. Forewings and hindwings hyaline, venation fuscous, stigma and scattered apical maculate markings on forewings and hindwings fuscous. Abdomen dorsally brownish ochraceous, ventrally paler, with numerous small fuscous spots.

Male genitalia: pygofer slightly broad, nearly rectangular, ventrally distinctly broader than dorsally (about 3.0:1) in lateral aspect (Fig. 12); posterior margin deeply excavated apically to accommodate anal tube, with a long, fingerlike, directed posteriorly process near apex in lateral view (Fig. 12); dorsal margin deeply excavated to accommodate anal tube, dorsal-lateral margins produced posteriorly in dorsal view (Fig. 13). Segment X (anal tube) narrow and elongate, with ratio of length to width near middle about 3.0:1; apical ventral margin protruded an angle on each side, apical dorsal margin deeply excavated to accommodate anal style in dorsal views (Fig. 13); epiproct relatively robust and long. Gonostyles large and broad, without spiniform setae on inner surfaces in basal half; narrow basally, broadest medially and reduced towards apex in lateral view (Fig. 12); upper margin with a small, obtuse process near upper middle, outer upper edge with a ventrally directed, hooklike process near middle in lateral aspect (Fig. 12). Aedeagus (Figs 14–16) large and symmetrical, with a pair of long and slender endosomal processes extended dorsally: basal 2/3 sclerotized and pigmented, apical 1/3 membranous; phallobase basally sclerotized and pigmented, with a pair of ventral angular lamellar processes which its edge membranous, without spine (Figs 15, 16).

##### Type material examined.

Holotype ♂ of *Awaramada fryeri* Distant, **[Sri Lanka]:** (1) Kandy, Ceylon, 7-02; (2) [red label] Type / H.T.; (3) [Distant’s handwriting] Awaramada  fryeri  Distant.

##### Other material examined.

**SRI LANKA:** 1♂, Ceylon, Udawattekelle, 1966.X.30, no collector; 1♂, Udawattekelle, Kandy, 1966.XI.10–13, no collector (both in USNM); 2♀♀, 1♂, [MNHN(EC)7458, 7459, 7460], Perad (=Peradeniya), Ceylan, Coll. Bugnion, Th. Bourgoin det. 1990; 1♂, Kandy, 7.02. Ceylon, Coll. Bugnion [MNHN(EC)7461], Th. Bourgoin det. 1990; 1♂, Kandy, 6.05. Ceylon, Coll. Bugnion [MNHN(EC)7562], Th. Bourgoin det. 1990 (all in MNHN).

##### Distribution.

Sri Lanka.

#### Genus Dorysarthrus

Puton, 1895

http://species-id.net/wiki/Dorysarthrus

Dorysarthrus Puton, 1895: 88; Melichar, 1912: 24; Metcalf, 1946: 29; Emelyanov, 1979: 16. Type species: *Dorysarthrus mobilicornis* Puton, 1895; by monotypy.

##### Remarks.

The genus *Dorysarthrus* was established by Puton in 1895 based on a single species, *Dorysarthrus mobilicornis* Puton, 1895 from Palestine. Now *Dorysarthrus* comprises four species, namely *Dorysarthrus alfierii* De Bergevin, 1923 (not ‘1924’ as stated by Metcalf 1946: 30, see De Bergevin 1923: 173), *Dorysarthrus mobilicornis*, *Dorysarthrus simonyi* Melichar, 1912 and *Dorysarthrus sumakowi* Oshanin, 1908, which are distributed in Egypt, Palestine, Syria, Aden, Arabia, Israel, Turkestan, Turkmen and Iran.

#### Dorysarthrus mobilicornis

Puton, 1895

http://species-id.net/wiki/Dorysarthrus_mobilicornis

Figs 2 5

Dorysarthrus mobilicornis Puton, 1895: 44; Melichar, 1912: 25; Metcalf, 1946: 30. Holotype ♀, Palestine (MNHN) [examined]

##### Type material examined.

Holotype ♀, **[PALESTINE]:** (1) [Puton’s handwriting] Dorysarthrus mobilicornis Put; (2) [Puton’s handwriting] Jerusalem; (3) ♀; (4) [red label] Type; (5) TH BOURGOIN det. 1990, [Bourgoin’s handwriting] Dorysarthrus mobilicornis PUTON, 1895; (6) MNHN-HF-90-106; (7) Museum Paris, MNHN(EH), 452 (MNHN).

##### Distribution.

Palestine, Syria.

### Subfamily Dichopterinae (Melichar, 1912)

#### Genus Dichoptera

Spinola, 1839

http://species-id.net/wiki/Dichoptera

Dichoptera Spinola, 1839: 286; Stål, 1862: 487; Kirby, 1891: 147; Kirkaldy, 1902: 50; Melichar, 1912: 41; Metcalf, 1946: 23. Type species:*Fulgora hyalinata* Fabricius, 1781; by monotypy.
Clonia Walker, 1858: 60. Type species: *Clonia lurida* Walker, 1858; by monotypy. Synonymised by Stål, 1962: 487.
Thanatophara Kirkaldy, 1904: 280. Nom. nov. for *Clonia* Walker.

##### Remarks.

The genus *Dichoptera* was erected by Spinola in 1939 as one of five dictyopharid genera for the family Dictyopharidae. A total of eleven species are included in the genus, which is restricted in the Oriental region. The genus *Dichoptera* was moved by Emeljanov (1979) from Dictyopharidae to Fulgoridae and a taxonomic review on this group is preparing (Song and Liang, in prep.).

#### Dichoptera hyalinata

(Fabricius, 1781)

http://species-id.net/wiki/Dichoptera_hyalinata

Figs 3 6

Fulgora hyalinata Fabricius, 1781: 315. Syntype[s] (?sex), Bangladesh [not examined].
Flata hyalinata (Fabricius): Germar, 1818:190.
Dictyophara hyalinata (Fabricius): Germar, 1833: 175.
Pseudophana hyalinata (Fabricius): Burmeister, 1835: 160.
Dichoptera hyalinata (Fabricius): Spinola, 1839: 289; Kirby, 1891: 133; Melichar, 1903: 18, Pl. I, Fig. 1; Distant, 1906: 238, Fig. 103; Melichar, 1912: 19; Metcalf, 1946: 25.

##### Material examined.

**INDIA:** 1♂, Chittoor, 1940.IX., P.S. Nathan (NCSU).

##### Distribution.

Bangladesh, India, Sri Lanka.

## Discussion

According to the diagnostic key and photos of the type species of the three genera *Pibrocha*, *Dorysarthrus* and *Dichoptera*, it seem obvious that *Pibrocha* may be more closely related to *Dorysarthrus* than *Dichoptera*. *Pibrocha* and *Dorysarthrus* share some synapomorphies from the following characters: the medium-sized species, much smaller and slenderer than *Dichoptera* species; the very elongate, nearly fractured cephalic process and a similar forewing venation. These distinct characters support well the monophyly of *Pibrocha* and *Dorysarthrus*, and they are assigned together in the subfamily Dorysarthrinae.

Emeljanov (1979) provided eighteen morphological characters for differentiating Fulgoridae from Dictyopharidae. Twelve of them and particularly the short crossvein in the clavus, support that Dorysarthrinae belongs to Fulgoridae. This character is also present in Cladodipterini (Melichar 1912; Metcalf 1946; Emelyanov 1983; Szwedo 2008; Song and Liang 2011; Bourgoin 2011). Thus, by transferring Cladodipterini to Fulgoridae from Dictyopharidae and elevating them to subfamily Cladyphinae (Cladodipterinae), (Emeljanov (1979, 2004, 2011) proposed to remove all Dictyopharidae with a claval cross vein to Fulgoridae, versus Melichar (1912), Muir (1930) and Metcalf (1946).

Urban and Cryan (2009) recently performed a first phylogenetic investigation of Fulgoridae based on DNA nucleotide sequence data from five genetic loci. In their phylogenetic analysis, these critical taxa were unfortunately unavailable for analysis. A more comprehensive study employing both molecular and morphological data is now needed, which will include the taxa identified by (Emeljanov (1979, 2004, 2011) as intermediate between Fulgoridae and Dictyopharidae.

In view of the problems of defining the distinctiveness between Fulgoridae and Dictyopharidae, Dorysarthrinae is tentatively preserved in Fulgoridae based on Emeljanov (1979) until further taxonomic and phylogenetic analyses in both families can be performed.

## Supplementary Material

XML Treatment for Pibrocha

XML Treatment for Pibrocha egregia

XML Treatment for Dorysarthrus

XML Treatment for Dorysarthrus mobilicornis

XML Treatment for Dichoptera

XML Treatment for Dichoptera hyalinata

## References

[B1] Bourgoin T (1993) Female genitalia in Hemiptera Fulgoromorpha, morphological and phylogenetic data. Annales de la Société Entomologique de France (Nouvelle Série) 29: 225-244.

[B2] Bourgoin T (2011) FLOW: Fulgoromorpha Lists On the Web, 1997-2011. Version 7, updated 2011-06-20. http://flow.snv.jussieu.fr/cgi-bin/entomosite.pl

[B3] Bourgoin T Huang J (1990) Morphologie comparée de l’appareil génital m le des Tropiduchidae Trypetimorphini et remarques phylogénétiques (Hemiptera, Fulgoromorpha). Annales de la Société Entomologique de France (N.S. ) 26 (4): 555-564.

[B4] De Bergevin E (1923) Descriptions of two new species Hemiptera Homoptera of Egypt. Bulletin de la Societe d’Histoire Naturelle de l’Afrique du Nord 4: 173-178.

[B5] Distant WL (1914) Some additions to the genera and species in the homopterous family Fulgoridae. Annals and Magazine of Natural History, London (8)13: 409–413.

[B6] Emeljanov AF (1979) The problem of differentiation of the families Fulgoridae and Dictyopharidae. Trudy Zoologicheskogo Instituta Akademii Nauk SSSR 82: 3-22.

[B7] Emeljanov AF (1983) Dictyopharidae from the Cretaceous deposits on the Taymyr Peninsula (Insecta, Homoptera). Paleontologicheskii Zhurnal 3: 79-85.

[B8] Emeljanov AF (1988) Order Homoptera. In: Ler PA (Ed) Keys to Insects of Soviet Far East. Vol. 2: Homoptera and Heteroptera. Nauka Publishing House, Leningrad. 496 pp. [U.S. Department of Agriculture, 2001, English translation.]

[B9] Emeljanov AF (1990) An attempt of construction of the phylogenetic tree of the planthoppers (Homoptera, Cicadina). Entomologicheskoe Obozrenie 69 (2): 353-356.

[B10] Emeljanov AF (2004) A new genus and tribe of Strongylodematinae (Homoptera: Fulgoridae). Zoosystematica Rossica 3(1): 52.

[B11] Emeljanov AF (2011) A new genus and species of lanterflies of the subfamily Cladyphinae (Homoptera, Fulgoridae). Entomologicheskoe Obozrenie 90 (1): 159-165.

[B12] Kirkaldy GW (1902) Memoirs on Oriental Rhynchota. Journal of Bombay Natural History Society 14: 46-58.

[B13] Liang AP (2000) Taxonomic notes on Oriental and eastern Palaearctic Fulgoroidea (Hemiptera). Journal of the Kansas Entomological Society 73 (4): 235-237.

[B14] Melichar L (1912) Monographie der Dictyophorinen (Homoptera). Abhandlungen der K. K. Zoologisch-Botanischen Gesellschaft in Wien 7 (1): 1-221, pls. 1–5.

[B15] Metcalf ZP (1946) General catalogue of the Hemiptera, Fasci. IV. Fulgoroidea, Part 8 Dictyopharidae. Smith College, Northampton, MA, 246 pp.

[B16] Muir F (1930) On the classification of the Fulgoroidea. Annals and Magazine of Natural History, London (10)6: 461473.

[B17] Nielson MW (1982) A revision of the subfamily Coelidiinae (Homoptera: Cicadellidae) IV. Tribe Coelidiini. Pacific Insects Monograph 38: 1318.

[B18] Song ZS Liang AP (2011) Taxonomic revision of the Oriental planthopper genus *Putala* Melichar, with description of a new species and resurrection of the genus *Avephora* Bierman (Hemiptera: Fulgoroidea: Dictyopharidae). Annals of the Entomological Society of America 104 (2): 154-170. doi: 10.1603/AN10069

[B19] Szwedo J (2008) A new tribe of Dictyopharidae planthoppers from Eocene Baltic amber (Hemiptera: Fulgoromorpha: Fulgoroidea), with a brief review of the fossil record of the family. Palaeodiversity 1: 75-85.

[B20] Urban JM Cryan JR (2007) Evolution of the planthoppers (Insecta: Hemiptera: Fulgoroidea). Molecular Phylogenetics and Evolution 42: 556-572. doi: 10.1016/j.ympev.2006.08.009 17011797

[B21] Urban JM Cryan JR (2009) Entomologically famous, evolutionarily unexplored: the first phylogeny of the lanternfly family Fulgoridae (Insecta: Hemiptera: Fulgoroidea). Molecular Phylogenetics and Evolution 50: 471-484. doi: 10.1016/j.ympev.2008.12.004 19118634

[B22] Yeh WB Yang CT Hui CF (2005) A molecular phylogeny of planthoppers (Hemiptera: Fulgoroidea) inferred from mitochondrial 16S rDNA sequences. Zoological Study 44 (4): 519-535.

